# Screening Potential Coating Materials to Reduce the Absorption of Volatile Phenols into Grapes During Simulated Wildfire Conditions

**DOI:** 10.3390/foods15091499

**Published:** 2026-04-25

**Authors:** Ignacio Arias-Pérez, Yan Wen, Arran Rumbaugh, Lik Xian Lim, Cristina Medina-Plaza, Anita Oberholster

**Affiliations:** 1 Department of Viticulture and Enology, University of California Davis, Davis, CA 95616, USA; yan.wen@dri.edu (Y.W.); lxlim@ucdavis.edu (L.X.L.); cmedinaplaza@ucdavis.edu (C.M.-P.);; 2 Instituto de Ciencias de la Vid y del Vino (ICVV) (UR-CSIC-GR), 26007 Logroño, Spain; 3 Desert Research Institute (DRI), 2215 Raggio Pkwy, Reno, NV 89512, USA; 4 Crops Pathology and Genetics Research Unit, Agricultural Research Service, United States Department of Agriculture, Davis, CA 95616, USA; arran.rumbaugh@usda.gov

**Keywords:** smoke taint, barrier spray, volatile phenols, glycosides, wildfires-climate change

## Abstract

Wildfires release volatile phenolic compounds (VPs) that can be absorbed by grapevines, potentially resulting in “smoke taint” in wines. This has emerged as a prominent issue for the global wine industry due to negative impact on wine quality and subsequent financial losses. Since effective vineyard mitigation strategies remain limited, this study evaluated the efficacy of different materials applied to grapes to reduce the absorption of smoke marker compounds under simulated wildfire conditions. Twelve materials were applied to individual Cabernet Sauvignon clusters close to harvest. Treated vines were exposed to intentional smoke using a purpose-built tent. Grapes from treated vines, as well as smoke-exposed and non-exposed controls, were harvested at commercial maturity. The results showed a strong stratification of VPs within the tent and in the grapes. Glycosylation began within hours of smoke exposure, with significant increases in almost all glycosylated compounds within 4 hours compared to non-smoked controls. Some materials reduced VP uptake relative to untreated controls (kaolin, charcoal, and two commercial coating formulations—GM3E and GMB6), whereas others increased the absorption of smoke-derived compounds (Parka and wipe-out). These findings highlight that those protective treatments may have variable and sometimes counterproductive effects on smoke compound uptake.

## 1. Introduction

Wildfires generate large amounts of smoke containing volatile organic compounds, particularly volatile phenols (VPs), derived from the pyrolysis of lignin and other plant components (e.g., grass, brush, and wood) [1]. These compounds, including guaiacol, cresols, and syringol derivatives [2,3], can be rapidly absorbed by grape berries and subsequently metabolized into glycosylated forms within the plant tissues [4,5]. Although glycosylation reduces their volatility, these conjugates act as latent precursors that can release free volatile phenols during fermentation [3], aging [6], or even in-mouth hydrolysis [7], ultimately leading to the perception of “smoke taint” in wines. This defect is characterized by undesirable smoky, ashy, and medicinal aromas, significantly reducing wine quality and marketability and causing substantial economic losses in affected regions [8].

In recent years, the increasing frequency and intensity of wildfires associated with climate change have intensified research efforts to better understand the mechanisms of VP uptake and mitigation strategies in vineyards and the resulting wines [9,10,11]. Several vineyard practices have been explored with varying degrees of success, including washing vines or grapes with water, aqueous ethanol, or other solvents [1,8,12,13,14]; misting grapes during smoke exposure [14]; partial defoliation of grapevines after or before smoke exposure [15]; harvesting grapes by hand [1,16]; or ozone fumigation of grapes [17]. While some treatments partially reduced the uptake of VPs by grapes, they did not prevent smoke taint, fueling the need for alternative approaches.

Among the proposed preventive strategies, the application of surface coatings or barrier sprays has gained attention as a potentially practical approach. These materials may act through different mechanisms, including the formation of a physical barrier, limiting the diffusion of smoke-derived compounds, adsorption or absorption of volatile phenols, and chemical interactions that reduce their bioavailability [18]. Numerous agricultural sprays and films have been evaluated: An anti-transpirant compound (acrylic polymer) reduced the uptake of smoke-derived guaiacol into grapes; however, this agrochemical product does not provide meaningful protection in recent studies [19]. On the other hand, the application of a film based on clay (kaolin) showed variable efficacy in preventing smoke-derived compound absorption, depending on the grape variety and treatment coverage [8,19,20]. A preliminary report suggested that applying hydrophobic or lipid-based coatings to grapevine provides protection [16]; however, earlier studies demonstrated that this treatment increased concentrations of VPs in smoke-exposed grapes [21]. Recent work on functional spray coatings has highlighted the importance of formulation design in mitigating smoke taint. For instance, cellulose nanofiber-based coatings incorporating chitosan and/or β-Cyclodextrin have shown potential for reducing volatile phenol uptake while maintaining grape physiological integrity [10]. Recent studies enclosing grape clusters in activated charcoal fabric prevented the absorption of VPs from the smoke [19,22]; yet this is not a financially viable option.

Given the limited effectiveness of current preventative measures in the vineyard, continued evaluation of commercially available or synthesized products for their ability to reduce the uptake of smoke compounds when applied to the surface of grapes is warranted. The objective of this study is to screen several commercially available products for their ability to act as a protective barrier against wildfire smoke in vineyards. Assessing their efficacy in reducing the uptake of smoke-derived VPs by grapes will provide guidance for future, more extensive vineyard trials. Ultimately, identifying an effective barrier spray to mitigate the impacts of smoke taint could enhance industry’s resilience to wildfires and help protect the livelihoods of grape-growers and winemakers.

## 2. Materials and Methods

### 2.1. Reagents, Solvents, and Standards

Chemicals and solvents of analytical and mass spectrometry (MS) grade were obtained from Sigma-Aldrich and Merck (Darmstadt, Germany). Volatile phenol standards and their glycosylates were sourced from different suppliers at the highest purities available from Alfa Aesar (Tewksbury, MA, USA), TCI (Portland, OR, USA), Sigma-Aldrich (St. Louis, MO, USA), and Toronto Research Chemicals (Toronto, ON, Canada). Deuterium-labeled standards were sourced from Toronto Research Chemicals (Toronto, ON, Canada), C/D/N Isotopes Inc. (Quebec, QC, Canada), and EPTES (Vevey, Switzerland). Ultrapure water used in this study was prepared in-house using the Super-Q water purification system (18 MΩ cm, Millipore Sigma, Bellerica, MA, USA).

The barrier products were sourced from the following suppliers: Parka™ Crop Cuticle Supplement from Cultiva LLC (Las Vegas, NV, USA); Surround^®^ WP (powdered kaolin) from Tessenderlo Kerley, Inc. (Phoenix, AZ, USA); EMP: GM-X1, EMP: GM-B6 and EMP: GM-3E from Gemm AG Products, LLC (Napa, CA, USA); Geosorb GR^®^ (oenological charcoal) and Microcol^®^ ALPHA (bentonite) from Laffort (Bordeaux, France); Chitosan from Spectrum Chemical Mfg Corp (New Brunswick, NJ, USA); *β*-Cyclodextrin from Tokyo Chemical Industry Co., Ltd. (Tokyo, Japan); and Wipe-out from R. L. Gibson (San Francisco, CA, USA). Detailed physicochemical information (e.g., composition, molecular weight, and particle size) were not available for most commercial products due to proprietary formulations. Available technical information and product classification are summarized in Supplementary Table S2.

### 2.2. Application of Coating Material to Grapevines

Four *V. vinifera* Cabernet Sauvignon vines grafted onto 420A rootstocks were selected as biological replicates for this study. Vines were grown at the Robert Mondavi Institute vineyards at the University of California, Davis, CA, USA (38°31′51.6′′ N; 121°45′16.0′′ W). The grape plant spacing was 2.1 m × 3.7 m in north–south oriented rows. The grapevines were planted in 2017 and trained to a lyre system.

Twelve treatments, plus smoked and non-smoked controls (Table 1), were applied to each of the four vines. For each treatment, three grape bunches per vine were selected and treated by submerging the entire cluster in the designated coating for 30 s to ensure uniform coverage. The coating materials were diluted with water to a concentration recommended by the manufacturer. The control bunches were submerged in deionised water for 30 s without the application of any coating material. Unsmoked control samples were also taken four rows away from the vines exposed to smoke. The treatment list is shown in Table 1. The different samples were collected in triplicate: (1) untreated and unexposed samples were collected prior to smoke exposure and 7 days after smoking experiments; (2) water-treated clusters exposed to smoke were collected at 4 hours, 1 day, 3 days and 7 days after smoke exposure; and (3) clusters treated with barrier spray and exposed to smoke were collected 7 days after smoking experiments.

### 2.3. Smoke Exposure

Three days after treatment application, the grapevines were exposed to smoke for 2 h in a rectangular tent structure (11 m long × 2 m high × 1.5 m wide) using polypropylene piping and polyethylene construction sheeting (Frost King, South Mahwah, NJ, USA) (Figure S1A). The smoke was generated by a wood pellet grill smoker (Z Grills, Ontario, CA, USA) using natural hickory pellets (Salt Lake City, UT, USA) for 1 hour. After active smoking, the vines were left exposed to the smoke for another hour within the tent. The smoker was connected to the tent enclosure through a flexible aluminum ducting (10 cm × 2.4 m) containing a fan (iPower, Duarte, CA, USA) (Figure S1B). A second fan was connected to a carbon filter (VIVOSUN, Ontario, CA, USA) at the output of the tent.

Air parameters were monitored within the tent using a portable air quality sensor, Thingy:AQ MK2 (Thingy IOT, Bellevue, WA, USA). The sensor was positioned in the midrow and monitored temperature (T), relative humidity (RH), ozone (O_3_), carbon dioxide (CO_2_), carbon monoxide (CO), volatile organic compounds (VOCs), and the concentration of particulate matter (PM1.0, PM2.5, PM4 and PM10).

**Figure 1:**
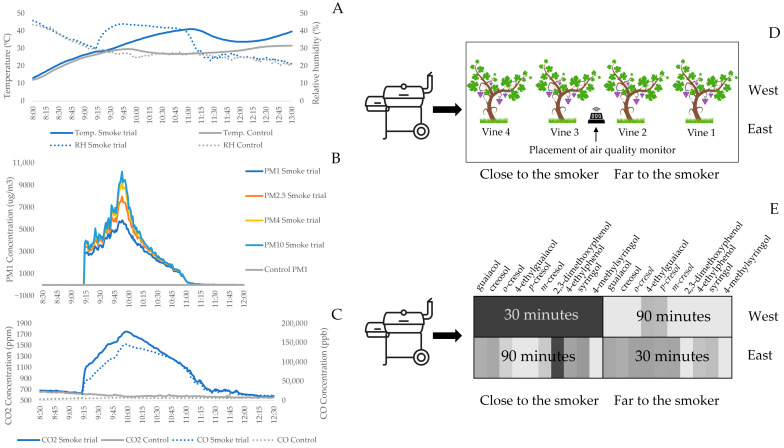
Air parameters measured during the field test by the sensor. Measurements taken on the day the smoke trial took place and the average of the experiment week are shown. (**A**) Temperature and relative humidity; (**B**) particulate matter (1.0, 2.5, 4.0,10); (**C**) CO_2_ and CO; (**D**) diagram of smoker, smoking tent, and vines; (**E**) heat maps depicting spatial variation in the VP levels in the tent during smoking. The color was determined through the atmospheric content of each compound. Black is the highest level and white is the lowest level. The presented data (relative area) were normalized through IS.

Atmospheric samples were collected during the smoke exposure at two time points. At 30 min, two samples were taken: one on the west side of the tent near the smoker and one on the east side of the tent away from the smoker. At 90 min, two additional samples were taken from the same locations (Figure 1). The samples were taken at the cluster height using 20 mL syringes and transferred into SPME vials from Restek Corp. (Bellefonte, PA, USA). The vials were immediately sealed and analyzed through headspace solid-phase microextraction coupled with gas chromatography–mass spectrometry (SPME-GC-MS).

Grapes were harvested a week after smoke exposure, including grape clusters that were not treated with coating spray and not exposed to smoke (Table 1). The grapes were harvested at 25.4 ± 0.7 ºBrix, pH 3.27 ± 0.1, 3.87 ± 0.02 g/L titratable acidity (tartaric acid), and a yeast assimilable nitrogen content of 97.7 ± 14.3 mg/L. Grape bunches were washed with water, dried with paper towels, and stored at −20 °C until the chemical analysis was performed in triplicate.

### 2.4. Chemical Analysis of Smoke-Derived Compounds

#### 2.4.1. Volatile Phenol Analysis of Atmospheric Samples

Free VPs from the tent were determined through headspace SPME-GC-MS using a method described by Cui et al. [9], with some modifications. Briefly, atmospheric samples were taken in SPME vials as described previously. The samples were spiked with deuterated internal standards (1 µL of 5 mg/L mixture solution). VPs in the headspace were preconcentrated on a CAR/PDMS fiber (Agilent Technologies, Santa Clara, CA, USA) and analyzed using GC-MS model 7890A (Agilent Technologies) via a Gerstel Autosampler (Version 1.2.3.1, Gerstel Inc., Linthicum, MD, USA). The GC Column was J&W DB-WAXetr (30 m × 250 μm × 0.25 μm) from Agilent. The sample was incubated and extracted with the fiber for 5 min at 50 °C. After the extraction, the fiber was desorbed in the injector at 220 °C for 10 min in the splitless mode. The oven temperature was initially held at 50 °C for 1.5 min and then increased to 220 °C at a rate of 8 °C/min, and finally at 30 °C/min to 250 °C and held for 5 min.

#### 2.4.2. Grape Sample Preparation

The grapes were thawed at room temperature, and all berries were removed from the rachis, and they were counted and weighed. All berries were homogenized using IKA ULTRA-TURRAX^®^ T18 (Staufen, Germany). The grape homogenate was used to analyze free VPs, acid-labile VPs, and VP glycosides in triplicate, as described below.

#### 2.4.3. Free and Acid-Labile Volatile Phenols of Grape Samples

Free VPs in grapes were analyzed by mixing CaCl_2_ (3 g) and MilliQ water (2 mL) with the grape homogenate (5 g) in a glass centrifuge vial. Internal standards of deuterated VPs were spiked to achieve 20 µg/kg, and homogenates were extracted with 2 mL of pentane:ethyl acetate (1:1). The mixture was vortexed for 30 s and incubated for 10 min, and the organic layer was collected in a 2 mL amber vial after centrifugation at 1578× *g* for 5 min.

Acid-labile VPs were analyzed by spiking 10 g of grape homogenate with deuterated internal standards (20 µg/kg) and transferring the mixture to PTFE tubes (VWR International LLC, Radnor, PA, USA). The samples were acidified to pH 1.00 ± 0.05 with concentrated HCl and incubated at 100 °C for 1 hour in a water bath. After hydrolysis, free VPs were extracted as described above.

The extracts were analyzed through GC-MS (Agilent 7890B), following a stable isotope dilution method reported previously [23]. The guaiacol, creosol (4-methylguaiacol), *o*-cresol, phenol, 4-ethylguaiacol, *p*-cresol, *m*-cresol, 2,3-dimethoxyphenol, 4-ethylphenol, syringol, and 4-methylsyringol concentration in the grape samples was quantified using calibration curves, with 2-methoxy-d3-phenol; 2-methoxy-4-methylphenol-3,5,6-d3; *o*-cresol-d7; *p*-cresol-d7; *m*-cresol-d7; 4-ethyl-d5-2-methoxyphenol; 4-ethylphenol-2,3,5,6-d4; and 2,6-dimethoxy-d6-phenol used as the internal standards.

#### 2.4.4. Volatile Phenol Glycoside Analysis of Grape Samples

The concentrations of VP glycosides were determined in berry homogenate by using solid-phase extraction (SPE) followed by the liquid chromatography-tandem mass spectrometry (LC−MS/MS) analysis according to methods reported previously [4], with some modifications. Each grape sample was analyzed in triplicate.

Prior to SPE, 5 g of grape homogenate was transferred to a 5 mL falcon tube (Fisher Scientific, Waltham, MA, USA). Internal standards of deuterated volatile phenol glycosides were spiked to achieve 50 µg/kg. The samples were centrifuged for 10 min at 4000 rpm and 4 °C. After centrifugation, 1 mL aliquot of the supernatant was extracted using the SPE method proposed by Caffrey et al. [4]. Strata-X solid-phase extraction cartridges were purchased from Phenomenex (Torrance, CA, USA). The analysis was performed using an Agilent 1290 Infinity ultra-high-performance liquid chromatography (UHPLC) system coupled with an Agilent 6545 quadrupole time-of-flight mass spectrometer (QTOF). The compounds (guaiacol-gentibioside, syringol-gentibioside, phenol-rutinoside, guaiacol-hexose, guaiacol-rutinoside, 4-methylsyringol-gentibioside, *p*-cresol-rutinoside, and 4-methylguaiacol-rutinoside) in grape samples were quantified using constructed calibration curves, with d3-guaiacol-beta-D-gentiobioside, d3-guaiacol-rutinoside, d6-syringol-O-beta-D-glucopyranosyl beta-D-glucopyranoside, d6-4-methylsyringol gentiobioside, d3-methylguaiaol rutinoside, d5-phenol-rutinoside, d7-*p*-cresol-rutinoside, and d3-guaiacol-beta-D-glucopyranoside used as the internal standards.

### 2.5. Data Analyses

The quantitative chemical analysis data was obtained using the Mass Hunter Workstation software (version 10.0, Agilent Technologies). All statistical analyses were performed using XLSTAT (2019, Addinsoft, New York, NY, USA), including one-way ANOVA with Fischer post hoc pairwise comparison (95%) test and correlation analysis (Pearson, 95% confidence level).

## 3. Results

### 3.1. Atmospheric Measurements

Figure 1 and Figure S1 depict the set-up of the smoking experiments. Temperature and humidity inside the smoking structure increased by 12.5 °C and 20% during the experiment when compared to the same time of day (Figure 1A). PM measurements (Figure 1B) and CO and CO_2_ concentrations (Figure 1C) were significantly elevated for the duration of smoke treatment. However, the VOC sensor was saturated and maxed out shortly after the smoker started at 500 μg/m^3^. The atmospheric samples inside the smoking structure demonstrate elevated levels of VPs, as well as a strong stratification in the distribution of VPs within the structure (Figure 1E and Table S3). Smoke density was higher in the first 30 min of the experiment and closer to the smoker.

### 3.2. Grape Volatile Phenol Composition

**Figure 2:**
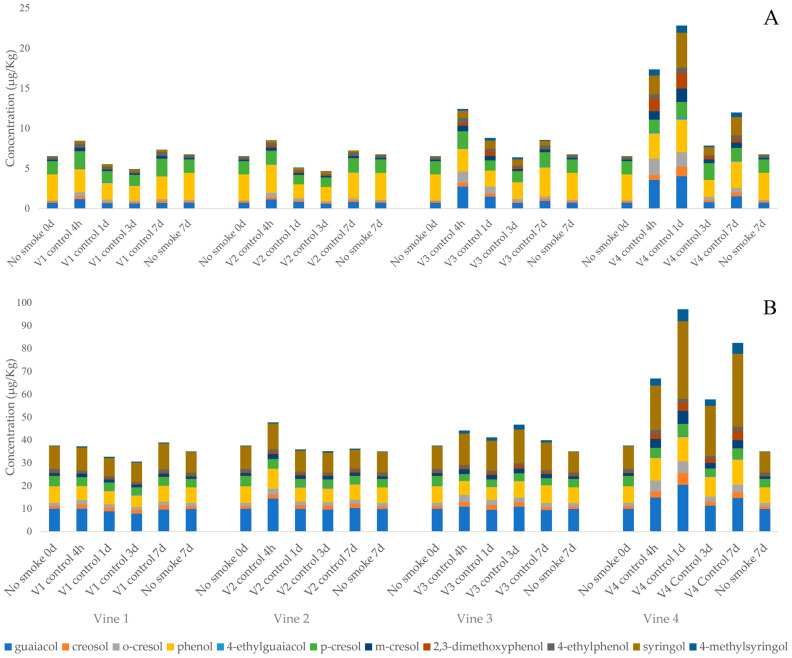
Concentration (μg/kg) of VPs found in the grape samples of free (**A**) and acid-labile forms (**B**). The figure shows the results obtained in the four vines (V1, V2, V3, and V4) at four different times after exposure to smoke (control 4 h, control 1 d, control 3 d, and control 7 d). The data from the non-smoked controls, before (no smoke 0 d), and 7 days after smoking (no smoke 7 d) are also presented.

Figure 2A and Table S4 demonstrate the heterogeneous distribution of smoke and the different degrees of VP uptake in each vine. Grapes from the vine closest to the smoker (vine 4) had significantly higher concentrations of VPs compared to grapes from the vine farthest from the smoker (vine 1), respectively. For example, the results 1 day after smoke exposure of the vine farthest from the smoker (vine 1) versus the nearest vine (vine 4) are as follows for acid-labile VPs—guaiacol: 8.81–20.4 µg/kg, creosol: 1.66–5.14 µg/kg, *o*-cresol: 1.45–5.08 µg/kg, phenol: 5.67–10.6 µg/kg, 4-ethylguaiacol: 0.28–0.40 µg/kg, *p*-cresol: 3.51–5.35 µg/kg, *m*-cresol: 1.40–5.70 µg/kg, 2,3-dimethoxyphenol: 0.42–3.69 µg/kg, 4-ethylphenol: 0.97–1.62 µg/kg, syringol: 8.00–33.9 µg/kg, and 4-methylsyringol: 0.46–5.20 µg/kg. Overall, free and acid-labile concentrations of VPs in grapes indicate a low level of smoke absorption. The main compounds contributing to acid-labile VPs (Figure 2B) are guaiacol and syringol. Since the grapes located on vine 4 were more impacted by smoke than grapes on the other three vines (as indicated by VP results in Table S4), the individual glycosides were only measured in grapes from vine 4 (Table 2). Syringol gentiobioside, 4-methylsyringol gentiobioside, and *p*-cresol rutinoside dominated the glycoconjugate pool analyzed. These observations are in agreement with the literature [24].

Notably, total and bound VP levels, as determined by acid hydrolysis, exhibited significant correlations with individual VP glycosides quantified by LC-QTOF-MS (Table S5).

### 3.3. Coating Treatment Efficacy

The heterogeneous distribution of smoke observed within the tent likely contributed to variability in VP uptake among vines and treatments. To effectively screen the ability of coating treatments to reduce VP absorption into grapes, only the most smoke-impacted vine was analyzed (vine 4). While this variability reflects realistic vineyard conditions during wildfire events, it also represents a limitation of the experimental design that should be considered when interpreting treatment efficacy.

**Figure 3:**
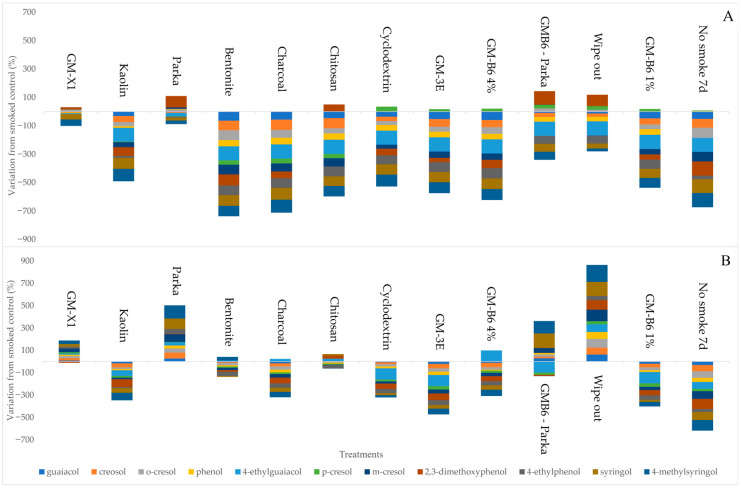
Relative variation (%) of VP concentrations with the different barrier spray treatments with respect to the smoked control 7 d. Free (**A**) and acid-labile forms (**B**) are shown. The figure shows the results obtained in vineyard 4 for all barrier sprays and the non-smoked control. Negative results are lower VP levels and positive results are higher VP levels than the smoked control sample.

Of the 10 surface coatings tested, most of them are commercial products used in horticulture for purposes such as pest and disease prevention, anti-transpiration, surfactants, and sunburn protection. In some instances, promising results were obtained, although these results should be interpreted cautiously. If only the free VP data is evaluated, most of the barrier sprays show some efficacy (Figure 3 and Table S6 in Supplementary Material), except for some compounds such as 2,3-dimethoxyphenol and *p*-cresol in some treatments. However, the same is not true for the acid-labile VPs, which include both the free and bound VPs. In this case, some treatments, such as those including Parka and wipe-out, show significant increases in smoke marker concentrations.

The order of the lowest to the highest concentrations of free VPs is charcoal < bentonite < chitosan < GMB6-4% < GM3E < no-smoke-7d < GMB6-1% < Cyclodextrin < kaolin < GMB6-Parka < wipe-out < GMX1 < Parka < smoked-control-7d. In contrast, the order of the lowest to highest concentration of total VPs is no-smoke-7d < GM3E < kaolin < charcoal < GMB6-4% < GMB6-1% < Cyclodextrin < bentonite < smoked control-7d < chitosan < GMX1 < Parka < GMB6-Parka < wipe-out. Not all individual VPs that were analyzed followed this trend. Barrier sprays such as GM3E, GMB6, charcoal, kaolin, and Cyclodextrin decreased the concentration of total VPs in the grape samples compared with the smoked controls (Table S6). It is important to focus on the acid-labile VPs when analyzing these results, because they will have greater long-term potential in terms of sensory and quality relevance than free VPs [6].

## 4. Discussion

### 4.1. Atmospheric Measurements

Elevated temperature and humidity during the smoking experiments and increased CO_2_ and CO concentrations within the tent could potentially modify plant physiology, such as stomatal conductance [1], potentially affecting compound uptake. PM measurements indicate a successful smoking exposure, reportedly higher than in other studies [13,14,19]. However, the results show that it is not a sufficient indicator of VP absorption by grapes. Although elevated PM levels were confirmed, their relationship with VP uptake is not straightforward. Particulate matter may serve as a carrier of semi-volatile compounds; however, the absorption of VPs by grape berries is primarily driven by gas phase diffusion and physicochemical interactions at the berry surface. Nevertheless, due to the spatial separation between the air quality sensor and the most impacted vines, as well as the heterogeneous distribution of smoke, direct correlations between atmospheric parameters and VP accumulation in grapes should be interpreted with caution.

### 4.2. Grape Volatile Phenol Composition

The stratification of smoke inside the tent and the differential uptake of VPs across vines aligns with previous research that has discussed the challenges of simulating wildfire smoke conditions and exposure to field-grown grapevines [19]. The low concentrations of smoke achieved in the tent could be attributed to hickory being used as a fuel source instead of barley, which most Australian researchers use [8,25]. Hickory pellets were used due their similarity in VPs composition to that produced during the 2020 wildfires in CA [26]. Furthermore, the phenological stage of the grapevine during smoking can play a pivotal role in the absorption of these compounds [27,28]; advanced phenological stages absorb less VPs in smoke [19,28]. Therefore, the absorption of these volatile compounds in the current study could also be reduced.

The results of VP absorption of the exposed vines show the same trend (Figure 2). In all vines, there is an initial increase in VPs, a subsequent drop at the 3-day time point, and an increase by day 7. This suggests the presence of intermediates or other metabolites of these VPs, as other authors have pointed out [9,11,21].

The main contributors to the total VP values were guaiacol and syringol. The maximum values found were 20.4 µg/kg and 61.9 µg/kg, respectively. However, some compounds increased whenever the vine was exposed to smoke, such as *m*-cresol and to a lesser extent 4-methylsyringol and 2,3-dimethoxyphenol, even when smoke exposure was very low. These compounds could serve as potential markers of smoke presence in the vineyard.

Similar to the acid-labile VPs values, it was observed that glycosylation begins to occur within a few hours of smoke exposure, with significant increases in almost all glycosylated compounds within 4 hours of smoke exposure versus the non-smoked control. The VP glycoside levels peaked between 1 and 3 days and reached an equilibrium between 3 and 7 days after smoke exposure. These findings are in agreement with other studies [13,20].

Total and bound VP data determined by acid hydrolysis showed significant correlations with individual VP glycosides determined by LC-QTOF-MS (Table S5). Although acid hydrolysis efficiency has been found to vary between compounds [24], there are clear correlations between individual bound VPs and acid-labile VPs analyzed. These strong correlations were also found in other recent studies with Muscat Gordo and Shiraz grapes [29].

### 4.3. Coating Treatment Efficacy

An important aspect in evaluating treatment efficacy is the distinction between free and acid-labile volatile phenols. While free VPs represent the immediately available fraction, acid-labile VPs include glycosylated forms that can be hydrolyzed during winemaking, contributing significantly to smoke taint perception. In this study, some treatments reduced free VPs without affecting acid-labile forms, suggesting limited effectiveness in mitigating long-term sensory impact. Therefore, the barrier spray results should focus on acid-labile forms.

To our knowledge, this study is the first to use GM3E, GMB6, and GMX1 as a barrier spray against grape smoke exposure. These are polymer emulsion/elastrometric polymers with essential oils that the manufacturer claims to be effective against fungal pathogens. Although two concentrations of GMB6 (1% and 4%) were investigated, the performance was irrespective of concentration (Figure 3 and Table S6). Interestingly, the results of the GM3E and GMB6 treatments are similar. Although, GM3 had similar concentrations of VPs compared unsmoked sample, and the spray was able to decrease guaiacol and 2,3-dimethoxyphenol by 69% and 66%, respectively. On the other hand, GMX1 did not reduce the levels of VPs with respect to the control sample.

Kaolin is a white, inert clay mineral, which forms a barrier film and acts as a broad-spectrum agricultural crop protectant for controlling damage from various insect and disease pests, acting as a growth enhancer and as a protectant against sunburn and heat stress [20,30]. This barrier spray consistently reduced the amount of VPs absorbed during smoke exposure by more than 53% and contributed to a reduction of 13 µg/kg syringol, which is in good agreement with the findings of other investigations. The kaolin treatment achieved reductions of 58–92% for most of the volatile phenol glycoconjugates in Merlot grapes exposed to smoke at harvest [20] and 20% reduction in VPs in Semillon grapes in a smoke model system [19]. In contrast, Culbert et al. [29] found that the application of kaolin did not provide significant protection in a model system. However, the conditions in these model systems are much more extreme than in the real vineyard situations because the grapes are exposed to high concentrations of VPs without the protection of the canopy and the metabolic alterations involved in removing the bunch from the plant.

The charcoal is an enological carbon from vegetal origin, which has been shown to be very effective at reducing the concentration of VPs and the intensity of sensory attributes associated with smoke taint in wines [31], and experiments in the vineyard have also achieved good results [19]. In the present study, the absorption of volatile phenols by grapes treated with charcoal (3%) and exposed to smoke was reduced by 55%. Likewise, Szeto and colleagues [19] showed that charcoal can prevent the uptake of up to 98% of the smoke-derived VPs observed in smoke-affected grapes enclosing grape bunches in activated carbon fabric. However, this treatment presents logistical problems in industrial application. Culbert et al. [29] demonstrated that the treatments based on carbon typically did not provide any significant protection to the grape against VP uptake in a model system. In addition, the carbon also could eliminate desirable aroma and color compounds, especially when added to wine [32].

β-Cyclodextrin is a cyclic oligosaccharide, which was found to be capable of removing VPs from wine [33]. This is the first time that it has been shown that Cyclodextrin could also be effective as a potential barrier spray, with reductions in VP levels of 22–100%, depending on the compound.

There are two products that produce similar results to the smoked control, bentonite and chitosan. Bentonite is a clay belonging to the sheet structured montmorillonite group with a high adsorption capacity, intended for protein stabilization. In the present study, grapes treated with bentonite showed a 27% reduction in VP absorption. This fining agent is widely used in wineries and it has already been tentatively used to mitigate the impact of vine exposure to smoke [31]. Chitosan is a polycationic β-1,4-linked-D-glucosamine polymer that forms a semipermeable film around plant tissues. It is already used as an effective biopesticide because it has antiseptic properties against unwanted microorganisms; it is also used to assist the action of fining agents. When chitosan was used as a barrier spray in this study, it prevented the uptake of some of the smoke-derived VPs observed in smoke-affected grapes, such as guaiacol and cresol derivatives. However, the vine application changes the aromatic and sensory profiles of the treated wines by affecting the nitrogen composition of the grapes [34].

Some treatments seemed to aggravate the adsorption of smoke volatiles, such as Parka and wipe-out. Parka is a patented blend of phospholipids (biofilm) designed to supplement the cuticle of growing fruit and foliage, and wipe-out consists of essential oils derived from different plants sources, used for crop management. In this study, Parka increased the adsorption of volatile phenols by 82% when applied exclusively and by 25% when the treatment was used in combination with GMB6. Wipe-out increased VP levels up to 142% in comparison to the smoked control. The results are in agreement with other investigations, suggesting that oily, hydrophobic materials may enhance the adsorption of VPs into grape berries [18,19,21,29].

The effectiveness of coating materials appears to depend strongly on their physicochemical properties and mode of action. Recent studies evaluating functional spray coatings have demonstrated that coating composition can significantly influence VP uptake through mechanisms including blocking, absorption, and adsorption [18]. Materials such as kaolin and charcoal likely reduce VP uptake through adsorption mechanisms, decreasing the availability of volatile compounds at the berry surface. Cyclodextrins may further reduce uptake through the formation of inclusion complexes with hydrophobic volatile phenols. In contrast, some polymer-based or lipid-rich coatings may enhance the partitioning of hydrophobic volatile phenols into the grape cuticle, facilitating their absorption [21]. Additionally, coatings may alter berry surface properties or plant physiological responses, further influencing compound diffusion and accumulation [10]. This may also modulate the relative proportions of free and acid-labile volatile phenols.

It is important to note that in the current study bunches were dipped in different barrier sprays and that the canopy was not treated. Under field conditions, much lower grape bunch coverage can be achieved with potentially lower efficacy [20,29]. Furthermore, the timing and frequency of application needs to be studied. Future studies should also aim to include detailed compositional and structural characterization of coating materials, as well as evaluate their performance under realistic vineyard conditions, to better understand the mechanisms underlying VP mitigation. Thus, no recommendations can be made until further field trials with barrier sprays that showed good efficacy have not been completed.

## 5. Conclusions

Wildfire smoke events are a significant challenge in winegrape growing regions around the world. Therefore, it is important to explore various strategies for managing the impact of grape smoke exposure, especially since it is unlikely that a single approach will fully mitigate the effects. This study provides a comparative evaluation of several commercially available coating materials for their potential to mitigate the uptake of smoke-derived volatile phenols in grapes under simulated wildfire conditions.

The experiment successfully simulated light to moderate smoke exposure within a controlled tent structure, which was confirmed by the presence of smoke-derived compounds in smoke, as well as an increase in PM during the smoking experiment. However, the distribution of smoke inside the tent was heterogenous, leading to various levels of VP accumulation across the vines. The results indicate that glycosylation of the VPs occurs rapidly, within hours, and that acid-liable VPs are well correlated to their respective glycoconjugate levels.

While certain treatments, such as kaolin, charcoal, and selected polymer-based formulations (GM3E, GMB6), showed promising reductions in VP accumulation, others increased the uptake of these compounds, highlighting the complexity of coating–smoke interactions.

Further work, including physicochemical characterization of coatings and validation under large-scale vineyard conditions, is required before practical recommendations can be made. Ongoing studies at pilot and industrial scale will help determine the feasibility of implementing these strategies in commercial viticulture.

## Figures and Tables

**Table 1:** Samples and treatment list evaluated for prevention of smoke taint in the vineyard.

Code	Treatment Dose	Typical Application
No smoke 0 d	No treatment	-
Control 4 h	Water	-
Control 1 d	Water	-
Control 3 d	Water	-
Control 7 d	Water	-
No smoke 7 d	No treatment	-
Parka	1% (*v*/*v*)	Cuticle supplement
Kaolin	60 g/L	Sunburn protectant
GM-X1	2% (*v*/*v*)	Pest management
GM-B6 4%	4% (*v*/*v*)	Pest management
GM-3E	1% (*v*/*v*)	Pest management
Cyclodextrin	25 g/L	Chemical scavenger
Chitosan	1% (*v*/*v*)	Biopesticide
Bentonite	1% (*v*/*v*)	Chemical scavenger
Charcoal	3% (*v*/*v*)	Chemical scavenger
Wipe-out	15 g/L	Crop management
GMB6-Parka	4% and 1% (*v*/*v*)	Pest management–cuticle supplement
GM-B6 1%	1% (*v*/*v*)	Pest management

Controls are smoked and harvested 4 h, 1 d, 3 d, and 7 d after smoke exposure (h = hours; d = days).

**Table 2:** Concentration values (μg/kg) of individual glycosides found in the samples of vine 4. The data was expressed as the average (*n* = 3) and standard deviation (SD).

	Guaiacol-Gentibioside	Syringol-Gentibioside	Phenol- Rutinoside	Guaiacol- Hexose	Guaiacol- Rutinoside	4-Methyl Syringol-Gentibioside	*p*-Cresol-Rutinoside	4-Methyl Guaiacol-Rutinoside
No smoke 0 d	<LOD	4.38 ± 0.97 c	0.88 ± 0.09 bc	<LOD-d	7.33 ± 3.02 a	<LOD-c	30.3 ± 1.01 c	<LOD-c
Control 4 h	<LOD	15.1 ± 0.94 b	1.25 ± 0.20 b	10.3 ± 0.07 b	6.88 ± 3.86 a	21.9 ± 0.08 a	34.1 ± 1.60 bc	0.56 ± 0.05 bc
Control 1 d	<LOD	29.6 ± 0.50 a	2.79 ± 0.36 a	19.2 ± 1.04 a	6.54 ± 2.14 a	29.6 ± 2.74 a	34.7 ± 3.86 bc	1.16 ± 0.19 a
Control 3 d	<LOD	28.9 ± 3.32 a	2.13 ± 0.44 a	11.9 ± 0.07 b	6.71 ± 2.44 a	24.6 ± 0.08 a	42.2 ± 3.36 a	0.73 ± 0.05 bc
Control 7 d	<LOD	31.8 ± 1.51 a	2.02 ± 0.20 a	9.34 ± 0.72 c	6.83 ± 3.27 a	24.5 ± 4.95 a	38.6 ± 3.23 ab	0.84 ± 0.34 ab
No smoke 7 d	<LOD	3.6 ± 0.20 c	0.63 ± 0.07 c	<LOD-d	5.70 ± 1.62 a	9.5 ± 9.85 b	31.9 ± 0.96 c	<LOD-c
*p*	-	<0.0001	<0.001	<0.0001	0.988	<0.001	<0.01	<0.001
LOD	0.29	0.60	0.37	0.83	0.33	0.35	0.38	0.33
LOQ	0.97	2.01	1.24	2.76	1.10	1.18	1.26	1.12

Different letters indicate significant differences (*p* < 0.05 according to pairwise Fisher test). Limit of detection (LOD). Limit of quantification (LOQ).

## Data Availability

The original contributions presented in this study are included in the article and Supplementary Material. Further inquiries can be directed to the corresponding author.

## Supplementary Materials

The following supporting information can be downloaded at: https://www.mdpi.com/article/10.3390/foods15091499/s1, Figure S1. Photograph showing Cabernet Sauvignon grapevines enclosed in smoke plastic tent before smoke exposure (A). Photograph showing the smoker used in the field experiment. The smoker was connected to the tent enclosure through a flexible aluminum ducting (B); Table S1. List of abbreviations; Table S2. Technical and commercial information of coating materials evaluated in this study; Table S3. Atmospheric content of volatile phenols during vine smoke exposure. Presented data (relative area) were normalized by IS; Table S4. Concentration values of volatile phenols compounds found in the non-smoked, control grapes and three treatments (Parka, Kaolin and GM-X1) of four vines. All expressed in micrograms per kilogram. The data was expressed as the average (among replicated measurements) and standard deviation (SD). Sample names: the letter V refers to the vine (1, 2, 3, or 4); treatment; and harvest time after smoking (4 h: four hours; 1 d: one day; 3 d: three days; 7 d: seven days). All treated samples were collected 7 days after smoking. Volatile phenols have been quantified in free (F) and acid-labile (A-L) forms; Table S5. Table of correlations between the VP glycosides (gg) and the respective concentration of free, acid-labile, and bound (acid-labile minus free) VPs in grapes. The data is given as Pearson correlation coefficient (R); Table S6. Concentration values of volatile phenols compounds found in the 12 treatments, non-smoked and control grapes of vine 4. Concentration values of volatile phenolic compounds found in control grapes and in all vine 4 treatments. All expressed in micrograms per kilogram. The data was expressed as the average (M) and standard deviation (SD). Sample names: the letter V refers to the vine (1, 2, 3, or 4); treatment; and harvest time after smoking (4 h: four hours; 1 d: one day; 3 d: three days; 7 d: seven days). All treated samples were collected 7 days after smoking. Different letters indicate significant differences (*p* < 0.05 according to pairwise Fischer test) for volatile phenol compounds among the samples in free (F) forms in capitols and acid-labile (A-L) forms in lowercase.
