# Supplementary material for: Screening Potential Coating Materials to Reduce the Absorption of Volatile Phenols into Grapes During Simulated Wildfire Conditions
Source: Foods. 2026 Apr 25;15(9):1499. doi: 10.3390/foods15091499 (PMC13164120; doi:10.3390/foods15091499)
Supplement: Supplementary file 1 [file foods-15-01499-s001.zip › foods-4241431-supplementary.pdf]

Supporting Information

Table S1. List of abbreviations

Abbreviation	Definition
CAR/PDMS	carboxen/polydimethylsiloxane
EMP	elastrometric polymer
GC–MS	gas chromatography–mass spectrometry
LC	liquid chromatography
LC−MS/MS	liquid chromatography-tandem mass spectrometry
LOD	limit of detection
LOQ	limit of quantification
PM	particulate matter
PTFE	polytetrafluoroethylene
Q-TOF	quadrupole time-of-flight mass spectrometer
RT	relative humidity
SPE	solid-phase extraction
SPME	solid-phase microextraction
T	temperature
UHPLC	ultra-high-performance liquid chromatography
VOCs	volatile organic compounds
VP-glycosides	glycosylated volatile phenols
VPs	volatile phenols

Table S2. Technical and commercial information of coating materials evaluated in this study

Product Name	Supplier	Product Type	Composition / Particle Size	Grade	Intended Use
Parka™	Cultiva LLC (Las Vegas, NV, USA)	Cuticle supplement	Proprietary polymer formulation	Commercial / agricultural	Anti-cracking / crop protection
Surround® WP	Tessenderlo Kerley, Inc. (Phoenix, AZ, USA)	Mineral coating	Kaolin (aluminium silicate)	Commercial / agricultural	Crop protection (sunburn, pests) /adsorptive barrier
EMP: GM-X1	Gemm AG Products, LLC (Napa, CA, USA)	Polymer coating	Proprietary	Commercial / agricultural	Pest management
EMP: GM-B6	Gemm AG Products, LLC (Napa, CA, USA)	Polymer coating	Proprietary	Commercial / agricultural	Pest management
EMP: GM-3E	Gemm AG Products, LLC (Napa, CA, USA)	Polymer coating	Proprietary	Commercial / agricultural	Pest management
Geosorb GR®	Laffort (Bordeaux, France)	Oenological charcoal	Activated carbon	Food grade / oenological	Wine fining / high adsorption capacity
Microcol® ALPHA	Laffort (Bordeaux, France)	Clay mineral	Bentonite	Food grade/ oenological	Protein stabilization / potential adsorption effects
Chitosan	Spectrum (New Brunswick, NJ, USA)	Biopolymer	Deacetylated chitin	Food grade	Antimicrobial / film-forming properties
β-Cyclodextrin	TCI Co., Ltd. (Tokyo, Japan)	Oligosaccharide	Cyclic glucose polymer / ~1 kDa	Analytical / food grade	Encapsulation / inclusion complex formation
Wipe Out	R. L. Gibson (CA, USA)	Surfactant-based	Proprietary	Commercial / agricultural	Crop management

Figure S1. Photograph showing Cabernet Sauvignon grapevines enclosed in smoke plastic tent before smoke exposure (A). Photograph showing the smoker used in the field experiment. The smoker was connected to the tent enclosure through a flexible aluminum ducting (B).

A.

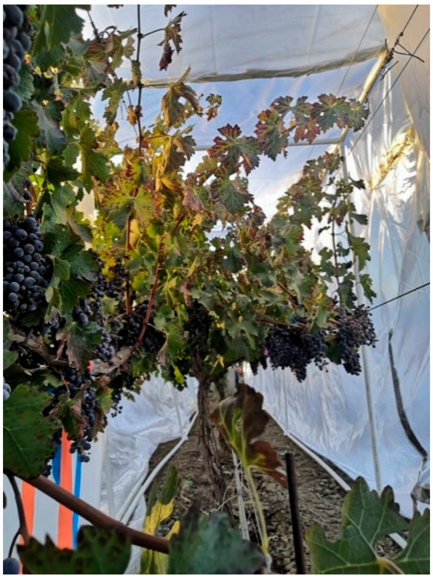

B.

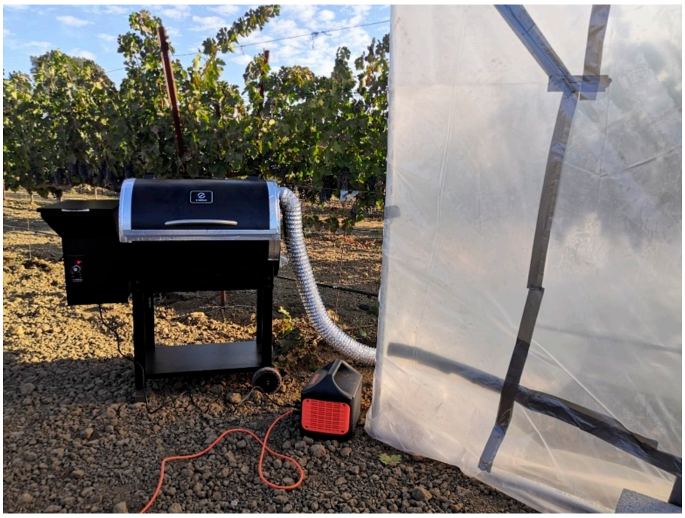

Table S3. Atmospheric content of volatile phenols during vine smoke exposure. The ratio of the individual volatile phenols contained in the atmospheric samples collected from inside the tent were determined by relative area (Individual VP area normalized by IS area).

**Sample**	guaiacol	creosol	*o*-cresol	phenol	4-ethyl guaiacol	*p*-cresol	*m*-cresol	2,3-dimethoxy phenol	4-ethyl phenol	syringol	4-methyl syringol
**30-1W**	2.772	0.269	0.282	3.191	0.037	0.266	0.446	0.028	0.117	0.010	0.054
**30-2E**	0.434	0.096	0.044	12.305	0.010	0.038	0.046	ND	0.006	0.002	0.000
**90-1E**	0.374	0.099	0.015	2.798	0.000	0.014	0.015	0.027	0.015	0.004	ND
**90-2W**	0.111	0.075	0.007	9.591	0.006	0.027	ND	ND	ND	ND	ND

Sample 30-1W: Sample taken at 30 minutes on the west side of the tent and close to the smoker. Sample 30-2E: Sample taken at 30 minutes on the east side of the tent and far to the smoker. Sample 90-1E: Sample taken at 90 minutes on the east side of the tent and close to the smoker. Sample 90-2W: Sample taken at 90 minutes on the west side of the tent and far to the smoker. ND: Not detected.

Table S4. Concentration values of volatile phenol compounds found in the non-smoked and control-smoked grapes of four vines. All expressed in micrograms per kilogram. The data was expressed as the average (among replicated measurements; n=3) and standard deviation (SD). Sample names: the letter V refers to the vine (1, 2, 3, or 4); treatment; and harvest time after smoking (4h: four hours; 1d: one day; 3d: three days; 7d: seven days). Volatile phenols have been quantified in free (F) and acid labile (A-L) forms.

Samples	Form	guaiacol		creosol		*o*-cresol		phenol		4-ethylguaiacol		*p*-cresol		*m*-cresol		2,3-dimethoxy phenol		4-ethylphenol		syringol		4-methyl syringol	
		Mean	SD	Mean	SD	Mean	SD	Mean	SD	Mean	SD	Mean	SD	Mean	SD	Mean	SD	Mean	SD	Mean	SD	Mean	SD
V1 Control 4h	F	1.18	0.047	0.34	0.018	0.52	0.023	2.85	0.14	0.057	0.003	2.20	0.082	0.46	0.020	<LOD	-	0.39	0.012	0.32	0.046	<LOD	-
	A-L	9.86	0.12	1.96	0.075	1.85	0.016	6.20	0.16	0.081	0.015	3.76	0.055	1.53	0.012	0.44	0.041	0.95	0.035	10.0	0.14	0.54	0.11
V1 Control 1d	F	0.69	0.038	0.15	0.002	0.28	0.004	2.06	0.031	0.13	-	1.36	0.015	0.21	0.005	<LOD	-	0.27	0.025	0.24	0.003	<LOD	-
	A-L	8.81	0.076	1.66	0.18	1.45	0.041	5.67	0.026	0.28	0.054	3.51	0.090	1.40	0.044	0.42	0.021	0.97	0.056	8.00	0.13	0.46	0.066
V1 Control 3d	F	0.65	0.003	0.15	0.010	<LOD	0.007	1.84	0.044	<LOD	-	1.32	0.016	0.17	0.001	<LOD	-	0.21	0.011	0.22	0.023	<LOD	-
	A-L	7.79	0.012	1.63	0.076	1.25	0.044	4.98	0.063	0.34	0.013	3.25	0.071	1.29	0.022	0.38	0.005	0.71	0.018	8.46	0.032	0.42	0.023
V1 Control 7d	F	0.71	0.009	0.28	0.006	<LOD	0.009	2.84	0.085	0.037	0.004	2.18	0.029	0.33	0.006	<LOD	-	0.48	0.035	0.25	0.022	<LOD	-
	A-L	9.60	0.11	1.91	0.128	1.57	0.006	6.88	0.16	0.083	0.014	3.79	0.064	1.52	0.035	0.52	0.020	1.23	0.050	11.4	0.097	0.37	0.046
V2 Control 4h	F	1.16	0.017	0.27	0.011	0.53	0.010	3.52	0.071	0.070	0.003	1.67	0.036	0.39	0.008	<LOD	-	0.36	0.004	0.28	0.014	<LOD	-
	A-L	14.4	0.42	1.79	0.050	2.44	0.056	8.79	0.23	0.082	0.001	4.19	0.093	2.22	0.043	0.81	0.034	1.12	0.016	11.3	0.29	0.61	0.039
V2 Control 1d	F	0.82	0.024	0.20	0.015	0.29	0.013	1.72	0.047	<LOD	-	1.16	0.052	0.23	0.011	<LOD	-	0.17	0.013	0.31	0.004	<LOD	-
	A-L	9.91	0.38	1.68	0.10	1.53	0.063	6.11	0.31	0.30	0.005	3.48	0.14	1.57	0.069	0.63	0.054	0.85	0.053	9.34	0.20	0.56	0.015
V2 Control 3d	F	0.61	0.018	0.14	0.014	<LOD	0.005	1.78	0.075	<LOD	-	1.18	0.035	0.18	0.002	<LOD	-	0.14	0.014	0.33	0.008	<LOD	-
	A-L	9.59	0.055	1.62	0.096	1.44	0.009	6.08	0.12	0.29	0.023	3.67	0.14	1.59	0.021	0.62	0.012	0.80	0.026	8.76	0.061	0.60	0.013
V2 Control 7d	F	0.83	0.019	0.24	0.008	<LOD	0.014	3.22	0.034	0.072	0.002	1.73	0.012	0.27	0.003	<LOD	-	0.42	0.005	0.23	0.034	<LOD	-
	A-L	10.3	0.012	1.98	0.075	1.51	0.009	6.72	0.075	0.073	0.002	3.70	0.056	1.52	0.031	0.90	0.010	0.84	0.006	8.13	0.020	0.55	0.046
V3 Control 4h	F	2.75	0.051	0.47	0.017	1.39	0.014	2.83	0.072	<LOD	-	2.13	0.038	0.68	0.011	0.39	0.015	0.55	0.010	0.95	0.065	0.23	0.006
	A-L	10.8	0.12	1.99	0.082	3.14	0.012	6.06	0.064	0.10	0.007	2.99	0.068	2.11	0.022	0.64	0.016	1.11	0.051	13.8	0.14	1.30	0.044
V3 Control 1d	F	1.51	0.12	0.38	0.007	0.86	0.019	1.98	0.066	<LOD	-	1.22	0.11	0.57	0.092	0.59	0.007	0.31	0.014	1.02	0.022	0.32	0.019
	A-L	9.49	0.069	2.07	0.12	2.26	0.044	5.62	0.048	0.29	0.087	3.02	0.062	2.02	0.025	1.02	0.011	0.89	0.026	12.9	0.074	1.48	0.051
V3 Control 3d	F	0.70	0.030	0.19	0.011	0.32	0.010	2.06	0.061	<LOD	-	1.33	0.010	0.25	0.006	<LOD	-	0.21	0.007	0.75	0.045	0.26	0.042
	A-L	10.8	0.044	1.96	0.091	2.01	0.010	7.14	0.027	0.22	0.022	3.22	0.052	2.10	0.008	1.51	0.023	0.72	0.023	14.8	0.091	2.13	0.12

Samples	Form	guaiacol		creosol		*o*-cresol		phenol		4-ethyl guaiacol		*p*-cresol		*m*-cresol		2,3-dimethoxy phenol		4-ethylphenol		syringol		4-methyl syringol	
		Mean	SD	Mean	SD	Mean	SD	Mean	SD	Mean	SD	Mean	SD	Mean	SD	Mean	SD	Mean	SD	Mean	SD	Mean	SD
V3 Control 7d	F	0.95	0.016	0.30	0.012	0.28	0.006	3.60	0.044	0.083	0.005	1.80	0.029	0.36	0.008	<LOD	-	0.40	0.010	0.55	0.004	<LOD	-
	A-L	9.36	0.10	1.34	0.021	1.76	0.021	7.69	0.52	0.085	0.006	3.08	0.030	1.76	0.017	0.83	0.009	0.85	0.041	12.1	0.056	1.10	0.026
V4 Control 1d	F	4.03	0.037	1.20	0.018	1.81	0.022	4.06	0.031	0.29	0.010	1.89	0.043	1.70	0.029	1.94	0.039	0.66	0.018	4.35	0.048	0.88	0.008
	A-L	20.4	0.27	5.14	0.15	5.08	0.032	10.6	0.084	0.40	0.019	5.35	0.10	5.70	0.027	3.69	0.065	1.62	0.047	33.9	0.19	5.20	0.13
V4 Control 3d	F	0.79	0.002	0.29	0.005	0.38	0.004	2.10	0.046	<LOD	-	2.10	0.32	0.46	0.002	0.39	0.023	0.17	0.019	0.97	0.010	0.21	0.004
	A-L	11.3	0.067	1.75	0.014	2.26	0.030	8.57	0.20	<LOD	-	3.31	0.062	2.42	0.046	2.05	0.028	0.93	0.013	22.0	0.31	2.73	0.063
V4 Control 7d	F	1.54	0.015	0.48	0.007	0.54	0.020	3.28	0.14	0.13	0.009	1.59	0.026	0.67	0.020	0.44	0.022	0.45	0.033	2.29	0.035	0.56	0.021
	A-L	14.5	0.35	2.65	0.016	3.15	0.036	11.1	0.072	0.27	0.023	4.64	0.051	3.55	0.037	4.04	0.13	1.74	0.031	32.0	0.42	4.72	0.19
No smoke 0d	F	0.74	0.020	0.19	0.003	<LOD	0.003	3.26	0.13	<LOD	-	1.63	0.019	0.20	0.002	<LOD	-	0.36	<LOD	-	0.009	<LOD	-
	A-L	9.93	0.11	1.18	0.092	1.41	0.015	7.24	0.22	0.12	0.009	4.43	0.061	1.24	0.016	0.39	0.009	1.35	0.038	9.95	0.035	0.31	0.045
No smoke 7d	F	0.74	0.013	0.18	0.013	<LOD	0.007	3.37	0.13	<LOD	-	1.68	0.015	0.22	0.003	<LOD	-	0.34	<LOD	-	0.008	<LOD	-
	A-L	9.79	0.18	1.18	0.065	1.39	0.023	6.88	0.064	0.10	0.005	3.65	0.024	1.11	0.025	0.37	0.012	1.27	0.032	9.01	0.094	0.28	0.013
LOD[a]		0.14		0.06		0.23		0.17		0.03		0.09		0.05		0.37		0.07		0.2		0.13	
LOQ[b]		0.43		0.17		0.69		0.52		0.09		0.27		0.15		1.12		0.22		0.6		0.4	

[a]LOD: Limit of detection; [b]LOQ: Limit of quantification

Table S5. Table of correlations between the VP glycosides (gg) and the respective concentration of free, acid-labile and bound (acid-labile minus free) VPs in grapes. Data is given as Pearson correlation coefficient (R).

Volatile phenols glycosides		Volatile phenols compounds		
		Free	Acid labile	Bound
guaiacol-gg	R	0.60	0.71	0.72
	p (t)	0.0089	0.0009	0.0008
syringol-gg	R	0.61	0.82	0.83
	p (t)	0.0072	0.0000	0.0000
phenol-gg	R	-0.09	0.61	0.80
	p (t)	0.7310	0.0074	0.0001
4-methylsyringol-gg	R	0.65	0.79	0.80
	p (t)	0.0036	0.0001	0.0001
p-cresol-gg	R	0.43	-0.25	-0.33
	p (t)	0.0734	0.3217	0.1846
4-methylguaiacol-gg	R	0.71	0.75	0.76
	p (t)	0.0010	0.0003	0.0002

Table S6. Concentration values (μg/Kg) of volatile phenol compounds found in the 12 barrier spray treatments, non-smoked and smoked control grapes of vine 4. The data was expressed as the average (M) and standard deviation (SD), *n*=3. Samples names: treatment and harvest time after smoking (4h: four hours; 1d: one day; 3d: three days; 7d: seven days). All treated samples were collected 7 days after smoking. Different letters indicate significant differences ($P < 0.05$ according to pairwise Fischer test) for volatile phenols compounds among the samples in free (F) forms in capitols and acid labile (A-L) forms in lower case.

Samples	Forms	guaiacol	creosol	*o*-cresol	phenol	4-ethylguaiacol	*p*-cresol	*m*-cresol	2,3-dimethoxy phenol	4-ethylphenol	syringol	4-methyl syringol
V4 Control 7d	F	1.54±0.015 B	0.48±0.007 A	0.54±0.020 C	3.28±0.14 AB	0.13±0.009 A	1.59±0.026 C	0.67±0.020 B	0.44±0.022 E	0.45±0.033 A	2.29±0.035 A	0.56±0.021 A
	A-L	14.5±0.35 d	2.65±0.016 d	3.15±0.036 d	11.1±0.072 d	0.27±0.023 bcd	4.64±0.051 d	3.55±0.037 e	4.04±0.13 c	1.74±0.031 d	32.0±0.42 f	4.72±0.19 e
V4 GM-X1	F	1.58±0.044 A	0.45±0.026A B	0.59±0.015 B	3.14±0.13 B	0.12±0.003 A	1.61±0.045 C	0.67±0.015 B	0.51±0.014 D	0.45±0.015 A	1.40±0.026 D	0.31±0.012 D
	A-L	15.8±0.46 c	3.15±0.069 c	3.79±0.037 c	12.4±0.16 c	0.30±0.042 abc	5.10±0.053 b	4.71±0.021 d	3.50±0.067 e	2.05±0.074 c	39.5±0.21 d	6.25±0.18 d
V4 Kaolin	F	1.06±0.023 E	0.27±0.011 D	0.39±0.008 D	2.93±0.063 C	<LOD B	1.55±0.032 C	0.43±0.018 D	<LOD G	0.39±0.005 C	0.50±0.014 J	<LOD I
	A-L	11.9±0.13 g	1.72±0.060 i	2.68±0.038 f	9.90±0.066 f	0.14±0.018 cde	3.99±0.19 fg	2.99±0.020 g	1.18±0.021 i	1.59±0.075 e	19.0±0.34 k	1.55±0.18 j
V4 Parka	F	1.58±0.019 A	0.44±0.015 B	0.60±0.014 B	3.42±0.23 A	<LOD B	1.54±0.034 C	0.74±0.013 A	0.80±0.028 B	0.42±0.016 B	1.83±0.078 B	0.42±0.024 C
	A-L	18.4±0.41 b	4.07±0.068 b	4.28±0.14 b	14.1±0.56 b	0.35±0.028 abc	4.79±0.085 c	6.02±0.17 b	4.16±0.037 c	2.52±0.091 a	61.9±0.25 c	10.3±0.15 b
V4 Bentonite	F	0.55±0.013 K	0.15±0.012F G	<LOD H	1.88±0.041 GH	<LOD B	1.11±0.021 D	0.21±0.005 H	<LOD G	0.15±0.019F G	0.56±0.012 I	0.15±0.014GH
	A-L	13.7±0.30 e	2.44±0.084 e	2.46±0.047 g	10.1±0.36 ef	0.29±0.025 bcd	4.11±0.026 ef	2.82±0.042 h	3.42±0.17 e	1.11±0.026 g	29.3±0.18 g	6.23±0.083 d
V4 Charcoal	F	0.67±0.002 I	0.13±0.013 G	0.23±0.009 G	1.79±0.027 H	<LOD B	1.04±0.016 D	0.29±0.008 G	<LOD G	0.15±0.010F G	0.36±0.009 K	<LOD I
	A-L	12.0±0.20 g	1.97±0.070 g	2.19±0.054 i	8.35±0.16 h	0.33±0.020 ab	3.99±0.13 fg	2.47±0.043 i	1.93±0.042 g	1.04±0.027 gh	20.6±0.31 j	2.47±0.032 h
V4 Chitosan	F	0.81±0.024 G	0.14±0.004 G	0.34±0.005 E	1.84±0.022 GH	<LOD B	1.11±0.006 D	0.28±0.003 G	0.66±0.014 C	0.14±0.012 G	0.73±0.009 G	0.15±0.011 FG
	A-L	14.3±0.079 d	2.58±0.058 d	2.92±0.012 e	10.3±0.050 e	0.33±0.049 ab	4.17±0.070 e	3.43±0.018 f	5.17±0.087 b	1.23±0.020 f	36.8±0.21 e	4.68±0.076 e
V4 Cyclo dextrin	F	0.97±0.032 F	0.34±0.009 D	0.39±0.043 D	2.00±0.17 FG	<LOD B	2.14±0.40 A	0.49±0.010 C	<LOD G	0.16±0.006 F	0.64±0.017 H	<LOD I
	A-L	13.5±0.39 e	2.19±0.038 f	2.52±0.062 g	9.22±0.21 g	<LOD e	3.92±0.078 gh	2.84±0.061 h	2.09±0.055 f	1.11±0.028 g	26.0±0.26 h	3.77±0.050 f
V4 GM-3E	F	0.72±0.011 H	0.22±0.006 E	0.35±0.008 E	1.99±0.023 FG	<LOD B	1.85±0.04 AB	0.37±0.005 F	<LOD G	0.14±0.010F G	0.63±0.011 H	0.13±0.003 H
	A-L	11.3±0.092 h	1.64±0.017 i	2.16±0.020 i	8.12±0.11 h	<LOD e	3.19±0.008 k	2.30±0.004 j	1.62±0.015 h	0.99±0.014 h	21.8±0.17 i	2.26±0.018 i
V4 GM-B6 4%	F	0.63±0.009 J	0.23±0.008 E	0.28±0.003 F	2.05±0.047 EF	<LOD B	1.92±0.015 A	0.37±0.004 F	<LOD G	0.13±0.001 G	0.56±0.014 I	0.13±0.009 H
	A-L	12.7±0.19 f	1.62±0.034 i	2.45±0.019 g	10.2±0.17 ef	0.36±0.010 ab	3.75±0.019 ij	2.53±0.034 i	2.20±0.052 f	1.10±0.034 g	19.3±0.028 k	2.08±0.029 i
V4 GMB6 –Parka	F	1.38±0.021 C	0.35±0.016 C	0.66±0.002 A	2.19±0.025 DE	<LOD B	1.99±0.034 A	0.68±0.009 B	0.87±0.013 A	0.20±0.008 E	1.02±0.010 E	0.24±0.013 E
	A-L	18.5±0.10 b	3.06±0.041 c	3.81±0.067 c	12.5±0.066 c	<LOD e	3.79±0.025 hi	5.11±0.012 c	3.65±0.038 d	1.69±0.010 d	74.0±0.44 a	9.93±0.074 c

Samples	Forms	guaiacol	creosol	*o*-cresol	phenol	4-ethylguaiacol	*p*-cresol	*m*-cresol	2,3-dimethoxy phenol	4-ethylphenol	syringol	4-methyl syringol
V4 Wipe out	F	1.33±0.014 D	0.37±0.013 C	0.61±0.014 B	2.23±0.013 D	<LOD B	1.99±0.017 A	0.66±0.007 B	0.80±0.010 B	0.20±0.003 E	1.49±0.092 C	0.45±0.008 B
	A-L	23.4±0.27 a	4.31±0.057 a	5.64±0.044 a	17.8±0.11 a	0.46±0.035 a	5.94±0.034 a	7.20±0.10 a	7.49±0.18 a	2.39±0.047 b	72.3±0.34 b	11.9±0.18 a
V4 GM-B6 1%	F	0.80±0.005 G	0.27±0.005 D	0.36±0.014 E	1.99±0.089 FG	<LOD B	1.87±0.005 A	0.41±0.007 E	0.28±0.005 F	0.16±0.001 F	0.83±0.031 F	0.17±0.006F
	A-L	11.4±0.22 h	1.82±0.069 h	2.34±0.038 h	9.23±0.10 g	<LOD e	3.25±0.078 k	2.48±0.041 i	2.16±0.046 f	1.04±0.028 gh	26.19±0.42 h	2.75±0.064 g
No smoke 7d	F	0.74±0.013 H	0.18±0.013 F	<LOD H	3.37±0.13 A	<LOD B	1.68±0.015 BC	0.22±0.003 H	<LOD G	0.34±0.015 D	<LOD L	<LOD I
	A-L	9.79±0.18 i	1.18±0.065 j	1.39±0.023 j	6.88±0.064 i	0.10±0.005 de	3.65±0.024 j	1.11±0.025 k	0.37±0.012 j	1.27±0.032 f	9.01±0.094 l	0.28±0.013 k
LOD		0.14	0.06	0.23	0.17	0.03	0.09	0.05	0.37	0.07	0.2	0.13
LOQ		0.43	0.17	0.69	0.52	0.09	0.27	0.15	1.12	0.22	0.6	0.4

[a]LOD: Limit of detection; [b]LOQ: Limit of quantification
